# Selections from Medical Works

**Published:** 1861-11

**Authors:** 


### SELECTIONS FROM MEDICAL WORKS.

*Prolapse of the Funis.*—Dr. T. Gaillard Thomas, in a lecture on the prolapse of the funis, published in the September number of the *American Medical Monthly,* speaks of the mortality of this complication, and gives the following results of the postural treatment of this accident.

" *Mortality.*—As far as the life of the child is concerned, there are few complications more dangerous than this, and certainly there is none which to the obstetrician brings more mortification and annoyance. Should convulsions or rupture of the uterus, with their very evident and terrible dangers, destroy the child, all can see and appreciate how such a result should naturally have occurred. But not so when an apparently insignificant part which can readily be returned to the uterus, has been displaced ; many a time will the practitioner doubt, lest he may have been wanting in mechanical ingenuity or activity of interference, and still more often will the bereaved parents and friends do so. The mortality of this accident is truly appalling. Of 355 cases of prolapse collected by Dr. Churchill, 220 children, considerably over one-half, were lost, and many of them after the mother's safety had been jeopardized by version or the application of the forceps. In 107 of these cases version (a most dangerous operation) was performed, and in 37 the forceps were applied.

*Frequency.*—With such a mortality, it is fortunate for the statistics of our art that this accident is not of frequent occurrence. To use the words of Velpeau, we may say that, " without being rare, prolapse of the funis is not very common," and we may have a definite idea concerning its comparative frequency by bearing in mind that it occurs a little oftener than transverse presentation. In the excellent statistical tables of Dr. Churchill, it is stated to have occurred 401 times in 98,512 cases, or about 1245½. in M. Schure, of Strasbourg, states it as 1 in 265 ; and Michaelis, of Kiel, as one in 282.

In the year 1856, I commenced some investigations into this subject, and proposed a plan by which I felt very positive that the difficulties attending the accomplishment of this indication might be overcome. It was not, however, until two years afterwards that I was able to obtain clinical evidence of the correctness of the theory ; and then having done so, I had the honor of reading an essay upon the subject before the Academy of Medicine of this city.

The philosophy of the proposed plan rested upon the facts which I now proceed to develop. You are doubtless aware that the axis of the uterine cavity, in advanced pregnancy, runs markedly from before backward and above downward ; that when the pregnant woman stands erect the fundus uteri tilts forward, and that the proper axis of the organ is in coincidence with a line running from the umbilicus (or a little above it) to the coccyx. Now place the woman on the back, and you will readily perceive that the organ offers to the slippery cord on every side an inclined plane, down which to slide into the vagina. This is likewise true when the woman lies on the side, though not quite in so marked a degree. The direction of the uterine axis then, and the lubricity of the cord, are the causes for the untoward results attendant upon the accomplishment of the first of the indications whch we have mentioned as an important one in treatment of this accident.

If by any means you could change the uterine axis so as to make it act in an opposite direction, it is evident that the slippery nature of the cord, so far from being detrimental, would be absolutely beneficial, and thus we would have overcome both the evil conditions with which we started out, and even made them useful to us.

Examine the diagrams given on the next page, and you will at a glance perceive that the desirable end may be accomplished by placing the woman on the knees and bowing the body forward upon the elbow, in the posture assumed by Eastern nations in devotion. By such a posture the axis is almost turned upside down, and the slippery cord will roll towards the fundus by the same law which brought the apple of Sir Isaac Newton to the ground.

During the last two years, I have not, I regret to say, industriously collected cases treated in this manner. Such as have been reported to me, I here exhibit to you by a table; guarding you, however, against the belief that a full *resume* of the results of the method are therein embodied. It will, at least, serve to show you that in many cases the method has been successful, and I own to the intensest gratification in thus recording the number of lives which have been saved by it.

| OBSTETRICIAN. | No. of Cases. | Failed. | Succeeded. | WHERE RECORDED. |
|---|---|---|---|---|
| Dr. Livingston, N. Y | 2 | 1 | 1 | To the Author. |
| " G. T. Elliot, " | 3 | 2 | 1 | " " |
| " Garrish, " | 1 | 1 | -- | Harveyan Circle. |
| " Vanderpoel, " | 1 | 1 | -- | To the author. |
| " P. Van Buren, " | 1 | 1 | -- | Academy of Medicine. |
| " Underhill, " | 1 | 1 | -- | Obstetric Section, Academy of Medicine. |
| " | 1 | 1 | 1 | To the author, by Dr. Bronson. |
| " Meminger, S. C | 1 | -- | 1 | " " |
| " J. R. Wood, N. Y | 1 | .. | 1 | Harveyan Circle. |
| " White, S. C | 1 | -- | 1 | Charleston Medical Journal. |
| " W. C. Rogers, Green Isl'd, N.Y. | 1 | -- | 1 | To the author. |
| " Worcester, N, Y | 1 | -- | 1 | " " |
| " Schmitt, N. Y | 1 | -- | 1 | " " |
| " Norton, | 1 | -- | 2 | New York Medical Press. |
| " Ferguson, N. Y | 2 | -- | 2 | To the author. |
| " Ramsbotham, London, | 2 | -- | 4 | Reported to Author, by Wm. Hall, Wilmington, N. C. |
| " Thomas, N. Y | 4 | -- | 3 | |
| " Brander, | 3 | -- | 1 | Cincinnati Lancet. |
| " Hawthorne, N. O | 1 | -- | 3 | To the Author. |
| " Furman, N. Y | 4 | 1 | 2 | " " |
| " McLeod, N. Y | 3 | 1 | 1 | " " |
| " Noeggerath, N. Y | 1 | -- | 2 | Contributions to Midwifery. |
| " Martin, | 2 | -- | 1 | American Medical Times. |
| " Woodhull, N. Y | 1 | -- | 1 | To the author. |
| " Elsberg, N. Y | 1 | -- | 1 | " " |
| " Bibbins, N. Y | 1 | -- | 1 | " " |
| " W. T. Brown, | 1 | -- | 1 | Cincinnati Observer. |
| " Mendenhall, Ohio., | 3 | -- | 3 | Cincinnati Lancet. |
| " A. K. Gardner, N. Y | 2 | -- | 1 | |
| Total | 47 | 10 | 37 | |

In justice to the method which I am now describing to you, (and which I have called the "postural treatment,") I must say that several of the cases of failure were due to the improper way in which it was followed out, or to some such complication as malpresentation, deformed pelvis, or placenta prævia. This, however, by no means applies to all."

